# Heterogeneity in Aging: Exploring Grip Strength Variability in Population-Based Age-Sex Cohorts

**DOI:** 10.1093/geroni/igaf122.2964

**Published:** 2025-12-31

**Authors:** Richard Fortinsky, Katherine Zavez, Lisa Barry, George Kuchel, Ofer Harel

**Affiliations:** University of Connecticut, Farmington, Connecticut, United States; University of Connecticut, Farmington, Connecticut, United States; University of Connecticut, Farmington, Connecticut, United States; UCONN HEALTH, Farmington, Connecticut, United States; University of Connecticut, Farmington, Connecticut, United States

## Abstract

Heterogeneity in aging is defined as variability in characteristics associated with chronological age. The concept of increased heterogeneity of health-related characteristics with increasing age has long been recognized as a tenet of geriatric medicine. However, a surprisingly limited amount of research has tested this concept. We used grip strength, an objective measure of physical function and frailty, to explore patterns of grip strength variability in population-based age-sex cohorts cross-sectionally and longitudinally. We constructed a study cohort from the UK Biobank database consisting of adults (52% female) with two measures of grip strength (n = 73,134). Age groups at initial measurement were: 40-44, 45-49, 50-54, 55-59, 60-64, and 65-69. In females and males separately, we compared: (1) patterns of grip strength variability across successively older age groups at one time point; and (2) changes over time in grip strength variability for individuals within the same age group cohort. Cross-sectional results revealed that, for females, grip strength variability did not differ significantly between pairs of successively older age groups; for males, we found statistically significant decreases in grip strength variability across successively older age groups. Longitudinal results showed that grip strength variability did not change over time within age group cohorts among females. In contrast, grip strength variability increased over time within nearly all age group cohorts among males. We conclude that heterogeneity in grip strength with aging operates differently for females and males, and that patterns of heterogeneity for males are strikingly different depending on whether age is explored cross-sectionally or longitudinally.

